# Rekindling of a Masterful Precedent; Bacteriophage: Reappraisal and Future Pursuits

**DOI:** 10.3389/fcimb.2021.635597

**Published:** 2021-05-31

**Authors:** Israa M. Abd-Allah, Ghadir S. El-Housseiny, Ibrahim S. Yahia, Khaled M. Aboshanab, Nadia A. Hassouna

**Affiliations:** ^1^ Department of Microbiology and Immunology, Faculty of Pharmacy, Ain Shams University, Cairo, Egypt; ^2^ Research Center for Advanced Materials Science (RCAMS), Advanced Functional Materials & Optoelectronic Laboratory (AFMOL), Department of Physics, Faculty of Science, King Khalid University, Abha, Saudi Arabia; ^3^ Nanoscience Laboratory for Environmental and Bio-Medical Applications (NLEBA), Semiconductor Lab., Metallurgical Lab, Physics Department, Faculty of Education, Ain Shams University, Cairo, Egypt

**Keywords:** bacteriophage, phage therapy, antibiotic resistance, phage proteins, phage pharmacotherapy

## Abstract

Antibiotic resistance is exuberantly becoming a deleterious health problem world-wide. Seeking innovative approaches is necessary in order to circumvent such a hazard. An unconventional fill-in to antibiotics is bacteriophage. Bacteriophages are viruses capable of pervading bacterial cells and disrupting their natural activity, ultimately resulting in their defeat. In this article, we will run-through the historical record of bacteriophage and its correlation with bacteria. We will also delineate the potential of bacteriophage as a therapeutic antibacterial agent, its supremacy over antibiotics in multiple aspects and the challenges that could arise on the way to its utilization in reality. Pharmacodynamics, pharmacokinetics and genetic engineering of bacteriophages and its proteins will be briefly discussed as well. In addition, we will highlight some of the in-use applications of bacteriophages, and set an outlook for their future ones. We will also overview some of the miscellaneous abilities of these tiny viruses in several fields other than the clinical one. This is an attempt to encourage tackling a long-forgotten hive. Perhaps, one day, the smallest of the creatures would be of the greatest help.

## Introduction

**Figure 1:**
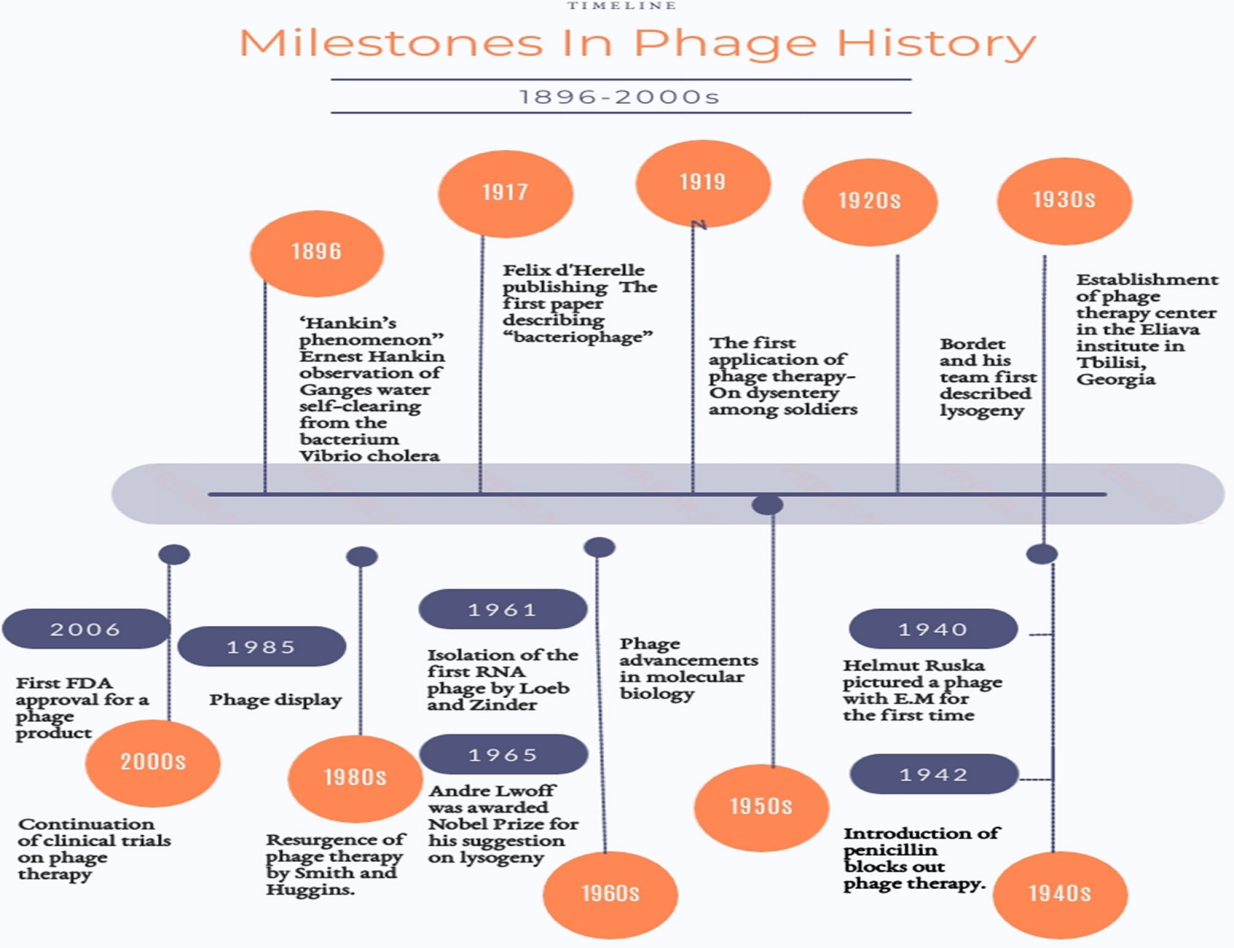
Timeline of the most considerable milestones since phage discovery.

With an approximate ubiquity ranging from 10^31^ to10^32^ (Carlton, 1999), bacteriophages (phages) are the least sophisticated yet most numerous biological entities on Earth. They serve as the greatest repository for genetic material. This is why they have been pictured as “the dark matter of the biosphere” (Hatfull, 2015). Technically, bacteriophages are viruses (Gordillo Altamirano and Barr, 2019) however, they are distinct from the viral archetype. Named after their inherent vocation, bacteriophages are obligate parasites that infect bacteria. They are their aboriginal destroyers. The earliest anecdotal news on bacteriophages is traced back to 1896 when the English naturalist, Ernest Hankin, published an article under the title of “The bactericidal action of waters of Jumna and Ganges on the Cholera microbe” (Abedon et al., 2011b) in which he described the absurd inexplicable observation of Ganges water self-clearing from the bacterium *Vibrio cholera* (Abedon et al., 2011b; Chanishvili, 2012; Nm, 2018). Back then, this was known as the’’Hankin’s phenomenon’’ (Chanishvili, 2012). Twenty years later, the formal chronicle of bacteriophage began. It is believed that phages were identified by Sir Frederick Twort in 1915 and separately two years later by the French-Canadian microbiologist Felix d’Hérelle who pursued his discovery by closely investigating the nature of these phenomenal agents (Chanishvili, 2012; Nm, 2018). However, obscurity has been always there, tainting the provenance of their discovery (Duckworth, 1976). Anyways, at least their appellation “bacteriophages’’ can be emphatically accredited to d’Herelle (Chanishvili, 2012). **Figure 1** shows some of the milestones in the history of phages (Gelman et al., 2018). Being natural parasites, they are most likely found wherever their hosts are amply present. Locales like sewage, wastewater, hospital vicinities, soil, human and animal bodies are always lush sources. They could be also found in extreme environments like marine water, ocean depths and the surroundings of low and high temperatures (Principi et al., 2019).

## Characters, Structure, and Classification

Bacteriophages are viruses that selectively utilize bacterial cells as their host. Being viruses, they are basically made up of a nucleic acid genome -either DNA or RNA, single or double stranded, encased into a cage of viral capsid proteins  (Kasman and Porter, 2020). They are mostly assembled as a simple build-up of head and tail. Despite this structure simplicity, bacteriophages are widely varied in their morphology, size and genomic make-up. For example, some have round, cubic or icosahedral heads that are joined to tails, others, though less common, are filamentous. Their sizes range from 30 to 110 nm (Weinbauer and Peduzzi, 1994). Regardless of their appearance, bacteriophages are non-motile and are transported only *via* Brownian motion (Kasman and Porter, 2020). Due to this high diversity, multiple classifications of phages are out there. For example, phages are divided according to the genetic material they harbor into four major categories: single stranded DNA phages (ssDNA), double stranded DNA phages (dsDNA), single stranded RNA phages (ssRNA), and double stranded RNA phages (dsRNA). Each of these groups is further classified into different families based on their morphology and the configuration of their genome; with dsDNA comprising the largest number: nine families, followed by ssDNA: two families. Relatively, DNA phages are better covered in literature, but limited reports are available on RNA ones. So, in few words, we are to spot some light on them. RNA phages comprise 2 families. On the dsRNA side: the family of *Cystoviridae.* Two years ago, a lytic *Cystoviridae* phage was isolated against *Streptococcus salivarius* and proved its potential for managing the infections it causes in the oral cavity (Cystoviridae, 2012; Beheshti-Maal, 2018). The other family is called *Levivirida*e which accommodates the ssRNA phages. ssRNA phages are probably the simplest of all identified viruses and their genomes range between 3500 and 4200 nucleotides. They are specific for Gram negative bacteria with pili. They all code for three proteins: coat, maturation and replicase. The coat protein builds up so that the capsid is composed of dimers. The maturation protein is the one responsible for the adsorption of the phage particle to the bacterial pili. The replicase protein: an RNA-dependent RNA polymerase that is used by the infected cells to synthesize RNA from the RNA template of the virion. ssRNA phages have been long utilized as study tools in the field of molecular biology because of their structure intelligibility. They were also used to learn about the process of protein synthesis and its regulation, and to closely examine each of the RNA replication, viral assembly and protein-RNA interaction. ssRNA phages have been applied in vaccine development, genetic drug delivery of small molecules, forensics and water quality control as well. Among the most researched of these phages are: Qβ, which served as the basis for understanding the RNA-dependent RNA replication in the early 1970s, and MS2: the one specific for F-pilus of *E. coli*, and the first living entity to be completely sequenced in 1976. A few MS2 phages with different lytic proteins can infect other organisms; namely, PP7 for *Pseudomonas aeruginosa* and AP205 for *Acinetobacter* sp. Along the last decade, the number of sequenced ssRNA phages has increased up to 15,000. In the larger context, some of them could be used therapeutically or industrially. However, this will require further elucidation of their protein structures and host interactions. Luckily, the cryo-EM has precipitated a breakthrough; it was possible for the first time to closely examine the ssRNA phage and view its genome within the capsid, revealing their 3D-structure (van Duin, 1988; Poranen et al., 2005; Uzan, 2009; Callanan et al., 2018; Zhang and Wu, 2020).

One more popular classification is the one bisecting bacteriophages into virulent and temperate ones. This is according to the way they interact with their natural counterparts; bacteria. Bacteriophages infect bacteria for survival, reproduction and completion of their life cycle. This life cycle is realized through one of two modalities; common in principal stages, but different in execution (Olszak et al., 2017).

### Lytic Cycle (Performed by Virulent Phages)

- Adsorption: After recognition of certain receptors on the bacterial cell wall or even other organelles such as sex pili and flagella, phages bind specifically to their hosts mostly using specialized attachment structures positioned onto their tails.
- Injection: Unlike most of the viruses, no uncoating occurs. Bacteriophages manage to insert their nucleic acid relying on some enzymes that drill through the bacterial cell wall (Olszak et al., 2017).
  For those attached to pili or flagella, the genome penetrates through these hollow pipes.
- Replication: Once invaded by nucleic acid, the bacterial macromolecule machinery is commandeered to synthesize phages’ structural protein components and replicate their nucleic acid.
- Assembly: The resulting phage coat particles assemble around the newly-formed nucleic acid molecules bringing about a quiverful of whole bacteriophages. In turn, these lurk inside the cells waiting for release and working for it.
- Liberation: Thenceforth, a two-step process led by phage-encoded enzymes, causes disintegration of the bacterial cell wall. Two protein groups are involved: holins and endolysins. Both together collaborate to form a lytic system that controls the timing of cell burst (Doss et al., 2017). First, holins act as a timekeeper; from inside the cell, they punch holes in the host’s cell membrane allowing the other protein group to make their way out towards the cell wall peptidoglycan. Endolysins; the second group, is composed mainly of: endopeptidases, glycosidases and amidases. Each subgroup is responsible for dissolving a particular peptidoglycan component. These endolysins in-turn and using their multi-enzyme power (Hodyra-Stefaniak et al., 2015) hydrolyze the bacterial cell wall armor, eventually leading to cell wall burst and release of nascent viral particles ready to attack other uninfected host cells, hence, perpetuating the cycle (Cisek et al., 2017).

### Lysogenic Cycle (Performed by Temperate Phages)

A temperate phage can adopt both lytic and lysogenic cycles depending on host availability, nutrient sufficiency for the host cells, phage concentration, and the presence of integration sites in host genome (Casjens and Hendrix, 2015). Among the most studied temperate phages are: The *E. coli* phages; λ and Mu, and the *Vibrio cholera* phage CTXphi. When incidents are conducive to lysogeny, both adsorption and injection occur. However, replication is not same as that of lytic cycle. Instead, the nucleic acid is integrated within the host genome through complementary attachment sites forming a “prophage” and starts to replicate in tandem with the host chromosome (Fogg et al., 2014; Howard-Varona et al., 2017). The same can also happen when host cells encounter stress conditions like unfavorable temperature and pH, a phenomenon known as “conditional latency”. As the name reveals, a prophage might wait for environmental recovery and then reverts back to the lytic cycle.

Needless to say, virulent phages are the advantageous ones when it comes to medicinal uses of phages. Nevertheless, the other type can be of great help when it comes to studying and understanding the latency embraced by some microbes as a life-sustaining strategy (Gandon, 2016).

Of relevance here is pseudolysogeny (quasi-lysogeny). It is defined as a temporary state of quiescence, in which the bacterial host cell does uptake phage’s genetic material, however, neither integration nor replication occurs. Nucleic acid inactively and independently resides within the cell (Ripp and Miller, 1997). This happens in response to host cell starvation, yet the phage would resort back to either of lytic or lysogenic cycles when the conditions resolve (Abedon; Fischer, 2019).

## Antibiotic Resistance: The Crisis

### Introduction

Antibiotic discovery is deemed an illustrious breakthrough in the recent history of medicine. Had it not been for antibiotics, most of the medical advances we are witnessing today, would have been doomed to failure. For instance, antibiotics held a space for successful operating on major surgeries, application of cancer treatment protocols and, organ transplantation. (Malik et al., 2017; Gordillo Altamirano and Barr, 2019).

Antibacterial resistance (ABR) can be roughly defined as the ability of bacteria to survive while exposed to concentrations of antibacterial agents they were once sensitive to, or to even higher concentrations, equal to those maximally achievable for therapeutic purposes (Patini et al., 2020). According to the reports of the Secretary-General of the United Nations (April, 2019), The World Health Organization (WHO) warns: because of antibiotic resistance, a rate of 10 million deaths per year could be reached by 2050 if the world doesn’t move appropriately towards effective interventions as declared by the O’Neill report in 2016  (Tangcharoensathien et al., 2017). For clarity, it was stated by WHO that: “Bacteria, not humans or animals, become antibiotic-resistant”. However, it may be of help if we figuratively address those suffering from resistant bacterial infections as “antibiotic-resistant patients” and the same goes for the infections caused by these pathogens. The idea can expand to defining antibiotic resistant environments like soils and rivers contaminated with traces of antibiotics. This is a useful tool to categorize them based on their different capacities to transmit antibiotic resistance (Hernando-Amado et al., 2019).

### Man-Made or Not?

For long, it has been argued whether only humans should be held responsible for the fulmination of antibiotic resistance or not. This has been soundly decided by WHO: “Antibiotic resistance occurs naturally, but misuse of antibiotics in humans and animals is accelerating the process”. As a matter of fact, developing resistance is a habitual trait inherent in bacteria, therefore, resistance emergence was inevitable. Surprisingly, some of the antibiotic resistance genes have been found into the antibiotic-naive microbial remnants present in permafrosts (D’Costa et al., 2011; Perron et al., 2015; Gordillo Altamirano and Barr, 2019). This provides a clue to the role of the natural antibiotics produced by bacterial cells as mediators of concerted communication within a bacterial community from the very beginning (Sengupta et al., 2013). This confirms that antibacterial resistance has been always there and is not just an emergent feature of the present day. Nevertheless, pursuant to the principle of tackling the root cause and not the effect; it is wise to impartially analyze the anthropogenic share to this onslaught, so that probably we can reach solutions. Foremost, the misuse of antibiotics for therapeutic purpose like underdosing, which is not enough to eradicate an infection but works perfectly well as a stress factor that challenges the bacteria for more resilience. Also, the superfluous prescription of antibiotics for non-bacterial infections is a blatant overdoing  (Gordillo Altamirano and Barr, 2019). In 2013, Center of Disease Control and Prevention (CDC) published the first report on the threats of antibiotic resistance to. It declared that up to 50% of all the antibiotics prescribed for human use are not needed or are not optimally effective as prescribed (CDC, 2020). In the same context, one cannot ignore the short-sighted use of antibiotics for augmentation of the livestock, animal farming and fish production. It has been estimated that two thirds of the total antibiotics used goes for this target. This might even eclipse the human use for clinical purposes (Done et al., 2015; Ventola, 2015). In addition, the heavy metals polluting the soil and water sources are now proved to have a role in horizontal gene transfer (HGT), hence, they help in the resistance-promoting process (Hsu et al., 2018; Jutkina et al., 2018). Furthermore, the hit-and-miss strategy followed in the disposal of pharmaceutical wastes, leakage of antibiotics into wastewater and the insufficient water treatment; all these contribute to the tenacious antibiotic dissemination in the environment and further exacerbate the problem, serving as a breeding ground for antibacterial resistance (Price et al., 2017). All these malpractices were not to constitute such a hazard unless they are backed up by the two sides of the original equation ruling the trajectory:

#### The Relentless Evolution of Bacterial Resistance Mechanisms

Bacteria are bizarre organisms. They spare no effort in order to maintain not only their survival but also their high-level fitness. Very few organisms possess the skill for exchanging certain genes among their different species through the so-called horizontal gene transfer. Among them, bacteria come genuinely first (Read and Woods, 2014; Lewies et al., 2019). The bacterial SOS response to certain kinds of stresses may trigger genetic mutations that, in the end, confer more resistance to antibacterial agents (Cirz et al., 2005). A few pieces of this bacterial weaponry include:

-Mechanical expulsion of the antibiotic molecules *via* efflux pumps.
-Remodeling the targets of antibiotics.
-Modification of certain biochemical pathways and the antibiotic target enzymatically rather than *via* genetic mutation, aiming for adaptation (Munita and Arias, 2016). For example, the efficacy of the aminoglycosides, the macrolides and the oxazolidinones is impeded by the enzymatic methylation of the bacterial rRNA. Another example is the modification of the vancomycin target chemically *via* conversion of L-serine to D-serine using the serine racemase enzyme (Wilson, 2014; Schaenzer and Wright, 2020).

#### Depletion of the Antibiotic Arsenal in the War Against Bacteria

The rate of development of novel antibiotic compounds is unwaveringly decreasing as revealed by CDC. Along the years between 1980–2000, more than 50 antimicrobial molecules were approved. Quite the reverse, less than 15 compounds were developed during the first millennial decade (Cirz et al., 2005). This was excellently depicted as the “ dry pipeline “of antibiotics (Gupta and Nayak, 2014; Luepke and Mohr, 2017) and can be attributed -most importantly- to the reluctance encountered by the researchers from the big pharmaceutical companies to invest in this direction. This perhaps is driven by the fear from the generally low return-on-investment of these products especially if they are not guaranteed to beat the odds. Also, some of these companies would prefer funding the drug products steadily demanded like those involved in treatment of chronic diseases.

### Consequences to Antibiotic Resistance

Some of the consequences that resulted from antibiotic resistance include:

-Prolonged hospitalization.
-Higher healthcare costs. The World Bank has warned that the economic loss caused by antimicrobial resistance globally may match the one caused by the 2008 global financial crisis and might reach a cost of $2 trillion every year by 2050 if no corrective actions are taken (Dadgostar, 2019).
-Previously-treatable infections may now be able to cause long-lasting disabilities and impairments. In turn, calamitous flow-on effects would ensue; going back to old, less-efficient infection control methods like isolation, debridement and amputation. Naturally, this compromises the individual’s quality of life (QOL) and places additional loads onto the community.
-Last but not least, increased rates of morbidity and mortality (Gordillo Altamirano and Barr, 2019; Lewies et al., 2019; Antimicrobial resistance, 2020).

These are just a tip of an iceberg, resistant pathogens have been even harnessed as a tool for bioterrorism, like *Escherichia coli* serotype: O157:H7 producing Shiga toxin, that can be used to cause hemorrhagic colitis epidemics if they contaminate food or water (Lewies et al., 2019).

Along the same line, a flagrant incidence from the medicinal archive obviously exemplifies the danger posed by antibiotic resistance and bears a strong lesson to learn. Nearly a century ago, particularly in 1918-1919, the deadliest pandemic in recent history had emerged. It was caused by an H1N1 virus, and was commonly known as the “Spanish flu”. It resulted in number of deaths estimated to be 50 million worldwide (Morens et al., 2008; 1918 Pandemic (H1N1 virus) | Pandemic Influenza (Flu) | CDC). Shockingly, compelling histological evidence revealed that most of these deaths were caused -in the first place- not by the virus itself but by its sequel: the secondary bacterial pneumonia. Since this scourge was long before the introduction of antibiotics, patients were helplessly left for death (Morens et al., 2008; Lewies et al., 2019). To one’s amazement, the more things change, the more they stay the same. Today, no stone is left unturned for what the whole world is witnessing because of the pandemic; coronavirus disease of 2019 (COVID-19) caused by severe acute respiratory syndrome coronavirus 2 (SARS-CoV-2). Till now, no one can decisively predict what the future for this virus is nor what complications else it might cause. In light of the above, today, more than ever, there is a dire need to seriously address antibiotic resistance. The response tactics selected to get around such a problem have to be multifaceted, inter-disciplinary and well-coordinated (Bartlett et al., 2013; Gordillo Altamirano and Barr, 2019). A sample of workable solutions could be: recruitment of vintage, no longer used antibiotics and tackling their downsides like chloramphenicol that was the cause of dangerous side effects such as grey baby syndrome, aplastic anemia, and bone marrow suppression (Shen et al., 2018). Other solutions include: devising a set of rules to be strictly followed when it comes to prescribing and dispensing of antibiotics, governmental partnering with multi-media centers to promote public health awareness in regard to proper use of antibiotics (WHO; Price et al., 2017; Gordillo Altamirano and Barr, 2019) and finally, allocating enough provision for investigating, exploring and testing promising compounds and encouraging the innovative non-compound approaches. Clearly, the crisis of antibiotic resistance is very labyrinthine with so many parties embroiled. It is useful to admit that at the present, there is no silver bullet that can deliver the death blow to such a problem. However, time is felicitous for recruitment of a practiced old-timer that holds the potential to be an adept member of the contemporary antibacterial materiel; bacteriophage.

A sizeable amount of knowledge regarding bacteriophages has been established along the past few decades. This succeeded to dispel a considerable part of the confusion hovering around these bacterial predators. So, this now, invites us to give these little bodies a second chance to restore some of the balance lost from the universal ecosystem and make use of them as auspicious antibacterial therapeutic tools.

## Assets and Shortcomings

### Assets

Highlighted here are some of the salient features of bacteriophages that favor their use therapeutically:

#### Specificity

Peculiarly, phages are characterized by unsurpassable host-specificity. However, this is determined by certain properties like the availability of phage receptors on the host cell surface, the bacterial defenses provoked against phages and the clonal diversity of the host species. This in-turn means narrow host range. If therapeutically administered, phages will attack only the pathogenic strain  (Hyman and Abedon, 2010) hence, absence of undesired off-target influences (Hyman and Abedon, 2010; Langdon et al., 2016). In addition to securing blindness to the human cells, this would make for two great therapeutic attributes. First, lower possibility of developing resistant bacterial mutants (Loc-Carrillo and Abedon, 2011), and second, no collateral damage can disrupt the untargeted bacterial flora normally harboring the human body (Skurnik et al., 2007; Abedon et al., 2011a). This differs from the side effects of antibiotics - particularly the broad spectrum ones -as they sometimes result in dysbiosis (imbalance of one’s natural microbiome) (Francino, 2015) and consequently occurrence of opportunistic infections like oral fungal candidiasis and the antibiotic-associated diarrhea (ABAD) that is caused by *Clostridium difficile.* However, this quality of phage is a double-edged weapon. Being that specific, phages can be used for treating an infection only after an industrious process of accurately identifying the etiological organism (Abedon et al., 2011a). This can be critical for quickly-progressive infections which necessitate rapid handling before complications arise. Fairly, this robs us of a privilege exclusive to the antibiotic regimens; the empiric therapy (Abedon et al., 2011a). But there is an outlet to this; genetically manipulating phages so as to express different tail fibers capable of binding to a larger number of variable hosts.

Another fix, is adjusting the phage isolation and enrichment procedures by using a variety of different bacterial host strains so as to induce the production of polyvalent phages rather than monovalent ones. Also, of benefit, is the application of “polyphage cocktail” preparations rather than the monophage ones. But this approach might hurdle the large scale phage production, because this will require more complicated isolation and formulation processes and subsequently, overtaxing manufacturing mandates and higher overall production costs (Gordillo Altamirano and Barr, 2019).

#### Self-Restraint

Phages, only reproduce where their target host is, and they are automated to go on actively replicating to the last bacterium present then, vanish, as if they are embracing a pattern of self-augmentation balanced out by self-restraining. This allows for some unique appurtenances:

- *In-situ* replication where the pathogenic strain is, thus limiting their influence only to the affected area (Abedon, 2011a).
-Single or few dose (s) administration is sufficient to maintain bactericidal effect till complete clearance of the infection. This could be considered a sort of active therapy (Payne and Jansen, 2000). Such self or “auto-dosing” (Rehman et al., 2019) spares the patient the need for repeated administration (Abedon, 2011a) hence, increases compliance and also helps in reducing treatment costs. Conversely, for antibiotics to be effective, they have to be maintained above their minimum inhibitory concentrations (MIC) which necessitates complex regimens of multiple dosing to make up for the drop that happens in concentrations between doses. Besides, the exposure of bacteria to sub-lethal concentrations between the doses provides them with a perfect opportunity to develop resistance (Carlton, 1999; Principi et al., 2019).
-Rapid clearance from the body once their host disappears, with no room for running wildly within the body, so minimizes the interaction with the immune system.

#### Bactericidal

One of the merits behind phages being bactericidal and not bacteriostatic is that the infected bacterial cells are irreparably destroyed. The case is quite the reverse when it comes to bacteriostatic antibiotics, as the exposed bacteria might be allowed to revel in resistance evolution.

### Shortcomings

In spite of these upsides, some key concerns regarding phage therapy are also present:

#### Anti-Phage Bacterial Resistance

Apart from specificity, let’s examine why a phage may succeed to destroy one host strain but not the other one. Starting with a question, are bacteriophages exempted from inducing bacterial resistance? As apparent, the answer is a no. For ages, bacteriophages have been rivals of bacteria. Opposing this, bacteria have got the effrontery to unfold a set of multi-front, anti-phage systems.

- **Altruistic suicide:** In order to protect the adjacent bacterial population, an infected cell may harness its toxin-antitoxin system to sacrifice itself and break the infection cycle in a phenomenon known as “abortive infection” (Abi) (Blower et al., 2012). Upon infection, a bacteriophage interferes with the antitoxin expression so that the toxin is now set free blocking the bacterial normal translation process and ultimately rendering the cell non-functional thus invalid for phage replication (Fineran et al., 2009).
- **Quorum sensing (a cell density monitoring mechanism):** For example, when infected by a virulent phage, *Pseudomonas* secretes a Pseudomonas Quinolone Signal (PQS) molecule so as to reduce swarming motility herewith, distancing and protecting the uninfected cell population. This is considered a way of intercellular communication (Bru et al., 2019).
- **Receptor modulation:** This occurs by altering the receptors that phages use for adsorption by one or more methods of the following:
  •Reducing the receptor availability *via* either loss of receptor or down regulation.
  •Masking them *via* secretion of an exopolysaccharide material to surround the cell
  •Conformational modification of the receptor 3-D structure (Labrie et al., 2010; Seed et al., 2012; Seed, 2015).
- DNA-oriented maneuvers:

Superinfection exclusion (SIE) systems are originally encoded by phages - the temperate ones -to protect a lysogen *(the bacterial cell after infection with a temperate phage)* from being infected by a different phage. Slyly, bacteria utilize the same to block viral DNA injection (Seed, 2015).

Restriction modification (RM) (Special for double-stranded DNA phages) systems are made of two parts (Sneppen et al., 2015): One is a restriction endonuclease (RE) enzyme that identifies any foreign unmethylated ds-DNA and damages it by cleavage. The second is a methyl-transferase enzyme that protects the indigenous bacterial DNA by methylating certain nucleotides (Stern and Sorek, 2011).

Clustered Regularly Inter-spaced Short Palindromic Repeats (CRISPR) and its associated proteins (Cas) is an RNA-mediated, sequence-specific defense mechanism possessed by 50% of prokaryotic genomes and play a pivotal role in protecting against phage attacks  (Stern and Sorek, 2011; Jakutyte-Giraitiene and Gasiunas, 2016). It acts as a genetic memory for those previously-encountered outsider genomes (Shapiro et al., 2018). It functions through a sequence of three steps:

Spacer acquisition/Adaptation: after injection of viral genetic material, its genome is detected and included as a spacer into the CRISPR cassette.

CRISPR RNA (cr-RNA) Formation: This newly composed CRISPR cassette is transcribed and further processed to form cr-RNA.

Interference: the invading viral DNA (and possessing a sequence complementary to that of the cr-RNA) is detected and ultimately broken down by a means of the “genetic scissors”; Cas proteins (Shapiro et al., 2018; Caflisch et al., 2019).

##### Co-Evolutionary Trade-Off

Bacteria and phages are tirelessly engaged in a co-evolutionary fray, and as anticipated, bacteriophages do not capitulate. On the contrary, they are always in the loop raising counter-attacks against most of their enemy’s attempts to hold them back. Since contrast enhances meaning, a little distinction from antibiotics is to be elucidated here. Antibiotics are chemical compounds; one can assert they are immutable static entities unable to put up with bacterial resistance. Whereas, phages are - conditionally - living agents; they have got dynamic systems capable of fighting back (Samson et al., 2013).

In response to bacterial resistance,

- Phages undergo genomic mutations to camouflage its DNA and so, it goes undetected by the bacterial restriction enzymes. Indeed, this happened with phages specific to certain *Bacillus* species by tweaking their restriction sites (Warren, 1980; Krüger and Bickle, 1983).
- Some phages are capable of dissolving the capsular material through the production of lipopolysaccharide-degrading enzymes (Samson et al., 2013).
- Phages may utilize a new receptor instead of the altered one like those already present for virulence (Filippov et al., 2011; Gómez and Buckling, 2011; Roach et al., 2013). For example, an *E.coli* population sensitive to a lambda phage exhibited resistance *via* down-regulating its binding receptor (LamB). In response, the lambda phage succeeded to exploit another receptor, the outer membrane porin F (ompF) to attach to its host cell, therefore, maintaining its infective ability (Meyer et al., 2012).
- Evading the abortive injection system, some phages have encoded for antitoxins to replace the bacterial ones (Samson et al., 2013).
- Confronting CRISPR/Cas, some evolved anti-CRISPR mechanisms (Pawluk et al., 2016).
- Some bacteriophages have developed a mutation so as to express an orphan methyltransferase enzyme that is uncoupled from the endonuclease counterpart and hence avoid the bacterial RM system (Samson et al., 2013).

Dazzlingly, a flip side to bacterial anti-phage resistance is perhaps out there. It is not necessarily deleterious. Quite the contrary, sometimes it can impose substantial fitness cost onto the organism in question. While evolving anti-phage resistance, a bacterium might end up simultaneously losing some of its virulence factors, or suffer from reduced growth rate and compromised ability to attack eukaryotic cells (Roach et al., 2013; León and Bastías, 2015). Remarkably, some bacterial strains were ripped off certain antibiotic resistant genes and regained their sensitivity to these antibiotics upon becoming phage-resistant (Chan et al., 2016). It is important here to mention that phages naturally outnumber bacteria with an approximate ratio of 10:1 (Hatfull and Hendrix, 2011). This emphasizes the boundless possibility to find a new phage active against a certain bacterial strain no matter how resistant it may have become this also draws attention to the untapped potential of phage cocktails as a way to overcome this antiphage resistance issue  (Regeimbal et al., 2016; Taneja and Kaur, 2016).

#### Immune Response

Generally, bacteriophages are safe with no major side effects reported. But phages are viruses, therefore they are capable of activating human host immune response. Human immune system is divided into two broad functionalities; innate and humoral. Innate immunity is primarily concerned with immune recognition *via* pattern recognition receptors (PRR). A bacteriophage might trigger it if they get recognized by the PRRs on phagocytes (Yen et al., 2017). An approach to overcome this is masking phages by connecting a non-immunogen like polyethylene glycol (PEG) to phage capsid proteins.

The other one, humoral (adaptive) immunity is mediated *via* a trove of various immune cells. Being a virus, a phage can induce a humoral response that ensues in the production of anti-phage antibodies (Biswas et al., 2002). This cannot be so problematic when dealing with acute infections as they last for just a few days and get eradicated before the production of these antibodies can even occur (Dąbrowska et al., 2014). However, for chronic infections and recurrent ones, this might be much of an issue since the antibodies produced would neutralize the phages and so, may affect the quality of the intended therapeutic outcome. A solution to this may be the administration of multiple low doses instead of single high one, yet, it is not that easy. Besides the previously mentioned regarding auto-dosing, a high “killing titer” of phages must be achieved in order to ensure the lytic effect. This possibly indicates the importance of the high dose approach. One of the solutions proposed is phage concealment by encapsulation (Kim et al., 2008).

Also, the immunogenicity of the phage preparation should be tested prior to trial on human subjects, so as to avoid any undesirable allergic reaction, as possible. A related worry is the liability of phages to induce an endotoxic shock. This, if any, could result from triggering the release of cytokines due to liberation of the bacterial cell wall residues after rupture occurs. In addition, rapid clearance of phages from circulation by the cells of reticuloendothelial system (RES) into lymph organs like liver, spleen and lymph nodes is another perturbation, especially for sequestered and systemic infections which require a reasonably maintained exposure to the anti-infective agents. The use of novel drug delivery systems like liposomes led to elongated circulation time. Anyways, one of the issues currently hindering the mainstreaming of phages in clinical practice is the lame understanding of phage interplay with immune system. Reports around this are limited, so, further investigation is necessary if phage is to be applied in therapeutics.

##### Allergic Potential

It is widely known that antibiotics may cause untoward allergic reactions in certain patients, especially: penicillin, penicillin-like drugs, tetracyclines and sulfonamides (Blumenthal et al., 2019). Though rarely tackled, one study has tested a polyphage preparation as a treatment for 12 patients allergic to antibiotics and suffering from wound infection. The result was similar to that of a control group receiving antibiotics. This spotlighted the low probability of phages to provoke an allergy.

##### Lytic Only?

When deciding on a phage for therapeutic purpose, only those strictly lytic should be considered. Lysogenic phages are not necessarily capable of timely bacterial lysis, so, they are not suitable for the desired outcome  (Carlton, 1999). Even among lytic phages, some are not good therapeutic candidates. Possessing an endonuclease enzyme is necessary to ensure the breakdown of bacterial genome before cell burst. This is imperative while choosing a therapeutic phage. Otherwise, this phage will play the role of a “superspreader” leading to persistence of intact chromosomes of a dead bacterial population in the environment which may aggravate the transmission of resistance genes by natural transfer (Keen et al., 2017).

##### Phage Genome

The genome of a bacteriophage selected for therapy should be well-characterized (Gill and Hyman, 2010), sequenced and screened for freedom from any of the genes coding for bacterial virulence factors (Cisek et al., 2017) phage-encoded toxins genes, antibiotic resistance genes (ARG) and lysogenic genes like integrase’s (Principi et al., 2019).

##### Mammalian Tissue Interaction

This aspect is a bit controversial. As implied earlier, bacteriophages almost lack for tropism in human cells. However, such a claim still requires an extraordinary evidence. Some phages can traverse tissue barriers like endothelium because of their suitable size, molecular weight and shape (Huh et al., 2019). This easily happens especially in inflammatory conditions in which the tight endothelial junctions widen. In addition, a recent evidence suggests the capability of phages to bind to certain receptors on human cells. This could be accounted for the resemblance between such receptors and the polysaccharides normally present on some bacterial hosts. For example, an *Escherichia coli* phage PK1A2 was successfully uptaken by neuroblastoma eukaryotic cells exhibiting polysialic acid moieties- a common bacterial capsule component (Lehti et al., 2017; Huh et al., 2019). That being said, still there is a knowledge gap in this area that would be a remiss if not thoroughly examined (Lawrence et al., 2019).

##### Regulatory Issues

Here comes the one of the sophisticated parts: absence of a well-defined body of the legislations and regulations essential to organize the use of phage products (either ready-made or not) and their position in the market (Bretaudeau et al., 2020).

The way to applying phage therapy is a long odyssey. But in an age of technology, an all-important solution to some of the phage challenges is genetic engineering.

## Genetic Engineering of Phages

### Introduction

Phages employed for health-related objectives are categorized into two entwined groups. This is based on the quintessence of the phage applied. Principally, we have untreated natural phages. After all the knowledge acquired through a time of full century, it is now no secret that these crude phages show some downsides. Today, living in the post-genomic era, we all have witnessed the breakthroughs presented in the overlapping fields of molecular biology, genetic engineering, bioinformatics and their applications in the area of biotechnology. So, it was not strange to try to make use of the genetic engineering techniques in order to fine-tune those rudimentary phages and harness them to achieve whatever target desired. This takes us to the astonishing world of genetically-engineered bacteriophages. It should be emphasized that genetic engineering of phages is conjured up not only to go about bacterial resistance but also as a way for vaccination, editing microbiome and drug delivery (Bretaudeau et al., 2020). Here, we will highlight some of the most recent phage genomic annotation tools and the primary difficulties faced during repeated attempts of phage therapy and how genetic engineering could tackle them.

### Genomic Annotation of Phages

After a genome is sequenced, its annotation is integral to make sense of such an array of nucleotides. The process of genome annotation is meant to spot all the functional genes along a genome and define what they are supposed to do. The same applies for phage genomes. Bacteriophage genome differs in nature from the bacterial one. Because of the limited capsid size, the genome it contains is usually compact, universally conserved genes are seldom found, the genes are smaller than their bacterial counterparts, and are frequently overlapping.

However, phage genome represents a great challenge for the bioinformatics tools currently in use. The softwares available like Glimmer, Prodigal, and GeneMarkS are validly used for bacteria and some viruses. They have been used to passably fulfill the purpose of phage genomic annotation. However, the accuracy of the results is hardly satisfactory, obviously, they were not originally tailored for the special nature of bacteriophage genomes. Lately, this has prompted the presentation of gene identification tools specific for phages. Some of the pioneering phage identification and annotation web servers are VirSorter, PhiSpy, and PHAST that was first launched in 2011. In the next few lines, we are to shed the light on two of the latest and most promising of them: PHASTER and PHANOTATE.

PHASTER (**PHA**ge **S**earch **T**ool **E**nhanced **R**elease): It is an upgrade of PHAST, and is mainly developed for prophage sequences present within bacterial genomes or plasmids that primarily confer resistance and pathogenicity on bacteria. It gets around some of the PHAST disadvantages, so it is faster, more efficient when dealing with large input queries, and allows for searching sequences gathered from metagenomic data (Arndt et al., 2016).

PHANOTATE: It is based on the postulation that non-coding phage regions are unfavorable and so, it deals with the phage genome as a network of multiple paths and then generates a weighted graph in order to discern the optimal path in which the open reading frames (ORFs) are more propitious, yet gaps and overlaps are disadvantaged (McNair et al., 2019).

Obviously, the cooperation between bioinformatics and software programming is still to provide us with more helpful tools.

### Difficulties Tackled by Genetic Engineering

#### Host Range Expansion/Spectrum Broadening

Assumedly, a bacteriophage appropriate for clinical use is the one effective against all the strains of one species without crossing the boundaries to another (Ross et al., 2016). To achieve this, two main strategies were implemented:

1. Alteration of the receptor binding proteins (RBP) usually present on the fibers of tailed phages. However, this is not yet widely applied (Citorik et al., 2014; Ando et al., 2015).
2. Facilitating cell accessibility (especially to those with external barriers like capsule and biofilm-producing bacterial cells). Some bacterial species like *Streptococcus pneumoniae*, *Haemophilus influenzae*, and *E.coli* are capable of producing a capsule around the cell to prevent detection by immune cells. Unfortunately, this can protect against phage penetration too. To overcome this, in one study conducted by Scholl et al., the gene of capsule polysaccharide-degrading K1-5 endosialidase enzyme was inserted into the genomic content of a T7 bacteriophage. This enabled the phage to penetrate through the capsule of *E.coli* and finally infect it (Scholl et al., 2005). Similarly, the gene construct of dispersin B, an exopolysaccharide (EPS)-degrading enzyme was incorporated in a T7 phage. This led to dissolution of the biofilm produced by *E.coli* population and subsequent invasion through the cells (Lu and Collins, 2007).

#### Prolonging Circulation Time

In the research done by Merril et al., they obtained a Lambda phage with a longer circulation time. After investigation, it appeared that the phage has undergone a mutation in one of its capsid proteins (Merril et al., 1996). Afterwards and by the means of genetic engineering, Vitiello et al. incorporated this very mutation into a different phage. He too succeeded to attain a long-circulating mutant (Vitiello et al., 2005).

#### Sequence-Specific Toxicity

Engineered phages can work as gene delivery systems. This way, a phage genome is to be manipulated with certain dominant genes so as to disrupt antibiotic resistance genes in targeted host cells (Yosef et al., 2015; Park et al., 2017). In the work performed by Vincent et al., *E.coli* populations resistant to both kanamycin and chloramphenicol were infected with M13 phagemid carrying sRNA silencing construct (Libis et al., 2014). As a result, considerable sensitivity was regained towards these two antibiotics. In the same way, a phage can deliver CRISPR/Cas9 sequences so as to rip the virulence genes away (Yosef et al., 2015; Park et al., 2017).This method should be especially helpful for infections caused by opportunistic organisms, such that virulence genes are warped, infection is cleared, yet normal flora is not affected (Kilcher and Loessner, 2019).

#### Phage Display

**Figure 2:**
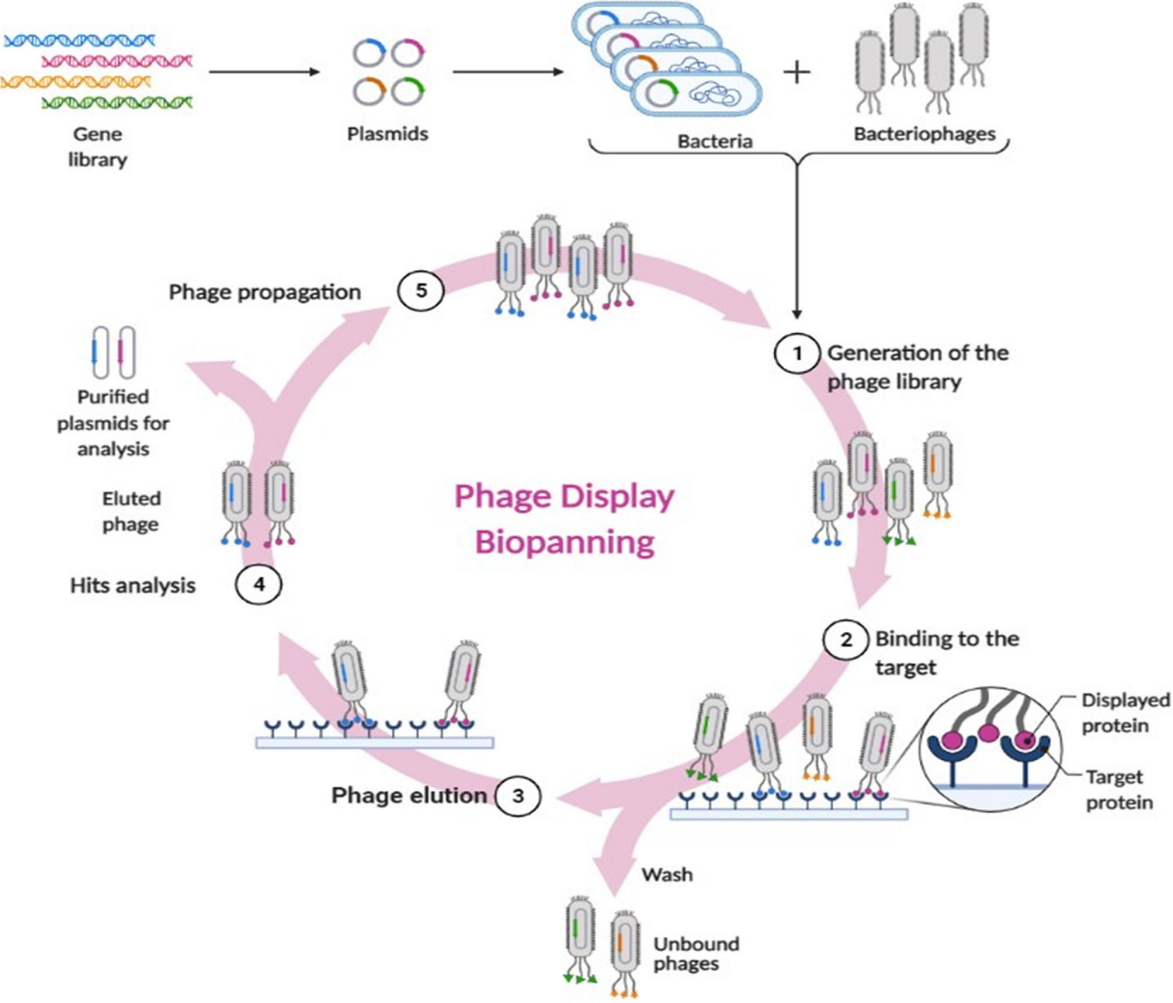
The main stages of the phage display biopanning cycle.

This is probably the most revolutionizing approach. It simply means genetic manipulation of phages so as to express antigens, peptides and antibodies on their coat surfaces as their own capsid proteins. This was first defined by Smith et al. in 1985 (Smith, 1985). Worthy of mentioning, that one half of the Nobel prize in Chemistry was jointly awarded to George P. Smith and Sir Gregory P. Winter in 2018 for their endeavors with phage display of antibodies and peptides (Saw and Song, 2019). These phages are able to treat intracellular infections by displaying antimicrobial peptides (AMPs) with cell-penetration ability (Staquicini et al., 2011; Rangel et al., 2012). The major contribution of phage display was actually in the area of vaccination. An antigen genetic make-up is firstly defined, then inserted for expression into phage genome. When the antigen is exhibited onto the viral surface, it can be efficiently traced by the immune cells (Aghebati-Maleki et al., 2016). Another major application of phage display is biopanning. It is the technique used to isolate and purify the peptides that are capable of specifically interacting with a foreign protein of interest. A random library of phages displaying certain peptides are incubated with the target protein. The unbound phages are washed away. The bound ones are those capable of identifying the protein of interest, hence, they are then eluted for purification, propagation and analysis (Stark et al., 2017; Lim et al., 2019). **Figure 2** depicts the main steps of biopanning.

#### Phagemid

As mentioned earlier, one of the concerns surrounding lytic phages is the endotoxic shock that may occur as a result of cell lysis, though no cases are reported so far (Reuter and Kruger, 2020). In anticipation, a special type of phages was contrived; synthetic phagemid. As shown by the name, it is composed of two parts: a non-replicating phage scaffold, wrapping up a plasmid. The plasmid genetic content is especially designed so as to be free from any proteins other than the main structural phage coat protein in addition to the desired display protein. In our case, a phagemid can code for antibacterial toxins or peptides (Qi et al., 2012). Once infection occurs, the plasmid would start replication and expression of these microbially-lethal substances eventually leading to bacterial cell death but without lysis (Krom et al., 2015).

### Phage Proteins and Enzymes

Perhaps phage-related proteins are going to be the next blockbuster in the world of infectious bacterial diseases, they might even outclass their genitors. One can expect them to be legally recognized as biologicals in the near future.

#### Biofilm and Phage Proteins

In the ever-lasting war between bacteria and its surroundings, cleverly, some could find haven in the scheme of collective security. Biofilm is a composite arena, where bacteria live in an architecture of multicellular communities embedded in a quilt of multiple components. In substance, it is formed of: extracellular proteins, polysaccharides, lipids and DNA (Slopek et al., 1983). Biofilm provides robust protection against the penetration of antibacterial substances, helps evading the immune system, promotes survival under unfavorable conditions (Briers et al., 2008)and substantially contribute to the persistence of chronic infections (Knezevic et al., 2011). Significantly, biofilms are main players when it comes to infections linked to surfaces, like catheters, medical devices (Leung et al., 2016), implants and prosthetics (Slopek et al., 1983). Biofilm-associated infections can be really difficult to cure. Thanks to its proteins, bacteriophage offers a way to manage such infections. Genomic content of phages codes for two major protein classes (Reuter and Kruger, 2020):

a. Virion-associated carbohydrate active enzymes, also known as, polysaccharide depolymerases. Their target is the external polysaccharides either in the biofilm matrix or the capsular one. They enable the phage to make an ingress to the receptors located on the bacterial cell wall and membrane. Finally, this fires the nucleic acid expulsion.
b. Peptidoglycan-targeting enzymes (Clark, 1962; Hubálek, 2003; González-Menéndez et al., 2018); these are subdivided into:
-Virion-associated lysins (VALs): They form localized pores into the peptidoglycan, eventually leading to translocation of the phage genome towards the bacterial cytoplasm.
-Endolysins: They settle in the cytoplasm, are well-timed and show up late towards the end of the life cycle. They reach back up to the peptidoglycan to intensively destroy it, eventually leading to cell lysis and rupture (Clark, 1962; Hubálek, 2003; Ma et al., 2008; González-Menéndez et al., 2018). In the upcoming years, phage proteins - especially the genetically optimized ones - shall occupy a prominent position in the therapeutics industry. A proof-of-principle is Artilysin, a product recently put on the market. It is unprecedentedly comprised of certain endolysins together with added peptides active against lipopolysaccharides (the impervious component of gram negative bacteria cell membrane) (Delbrück, 1940; Singla et al., 2015; Chadha et al., 2017; Leung and Weitz, 2017). This unique combination merged two different features: bactericidal effect of endolysin plus permeation enhancement activity of the peptides. Not to mention, endolysins advantageously keep the specificity of their parent phages, and as proclaimed by Gerstmans et al. (Chadha et al., 2017), Artilysin is capable of eradicating the persister cells (dormant cells that are randomly generated within a biofilm and are imperturbable when exposed to antibacterials) (Abedon, 2011b). Art-175 is an artilysin product which proved effectiveness against some recalcitrant strains of *Pseudomonas aeruginosa * (Vandenheuvel et al., 2015). Artilysins could surpass both antibiotics and even the use of whole bacteriophages because these inevitably require metabolically active and highly replicating host cells in order to function (Slopek et al., 1983). This might be a dismissive trait of bacteriophages, since it can possibly lead to self-cessation and ultimately, failure of therapy as the number of actively replicating cells declines  (Singla et al., 2015). Plausibly, a lot of work is still to be done regarding this family of proteins. For example, fine adjustments of active domains, and the creation of “chimeolysins” which are defined as “the assembly of multiple enzymatic domains from various lytic endolysins into one chimeric proteins” so as to improve their physical properties like thermal stability and solubility, besides increasing their efficacy (Slopek et al., 1983; Donlan and Costerton, 2002; Davies et al., 2007; Abedon, 2011a; Vandenheuvel et al., 2015; Abdelkader et al., 2019; Jault et al., 2019; Lin et al., 2019). In the work performed by Wang et al., a cell-penetrating peptide (CPP) usually used for ease of access to human cells, was linked to an endolysin purified from a *Staphylococcus aureus* specific phage. Unrivalled, this can be used for treatment of intracellular staphylococcal infections (Scoffone et al., 2017). In the same regard, the first human therapeutic product of an endolysin, “Staphefek”, indicated for the management of *Staphylococcus aureus* skin infections is now commercially available (Abdelkader et al., 2019). Now, it is quite tangible why this territory of genetic engineering well deserves a description of “The art of lysing”.

### Phage Engineering: A Gateway for Renown?

It is believed that, should pharmaceutical companies realize the unique qualities of genetically-engineered bacteriophages and the ensuing possibility of their high return-on-investment, they would probably venture to fund the development of such products. Also, this may create a competitive realm where each company would compete to seize the chance of an intellectual property (IP) protection right for these novel products. This cannot be the case with pristine phages. They have been known for years and are widely available; leaving not much room for patency seeking (Kilcher and Loessner, 2019).

## Phage Pharmacotherapy

As is well known, pharmacotherapy leans on two pillars: pharmacodynamics and pharmacokinetics. Here, we will highlight some of the central aspects of phage pharmacotherapy.

Pharmacodynamics, the response of the body to a drug, is roughly split into terms of efficacy and safety. Phage efficacy is most importantly described as the ability of a phage preparation to completely eradicate or at least decrease the count of bacteria causing certain disease with the purpose of restoring the previous health state or alleviating some of the manifestations caused by this disease. As for safety, besides those introduced earlier, here are a few additional emphasis points:

-In spite of all the still need-to-be answered questions concerning phage immunology, so far neither a serious reaction nor an idiosyncrasy has been described.
-Since safety’s flip side is toxicity, phage stocks intended for therapeutic use must undergo a profound purification process to ensure preclusion of any bacterial debris. Purification degree required differs according to the route of administration intended. For invasive ones like intravenous, intraperitoneal and intra-articular injection, high purity must be secured by means of ultracentrifugation or ultrafiltration. Whereas, for more customary routes like the oral and topical ones; high-speed centrifugation, precipitation using PEG and filtration will sufficiently do.
-One last point to reemphasize is the stringent selection of virulent phages and the obviation of temperate ones-unless genetically manipulated for a certain purpose. Both types may manifest transduction, and the genes transferred to another bacterial cell are usually harmful. However, it is most troublesome with temperate phages because the genes transferred will stay around since cells themselves will. On that account, genomic characterization of therapeutic phages is a prerequisite (Briers et al., 2008; Knezevic et al., 2011).

With respect to pharmacokinetics, phage kinetics halves into two branches. The first, depicts the typical meaning of pharmacokinetics: the impact of the body on the drug as represented by absorption, distribution, metabolism and elimination (ADME). In our context, both phage count and activity are the concern. These proved to be considerably affected by factors related to formulation, route of administration and target tissue (Slopek et al., 1983).Though most routes have been tested and are in-use, some could outdo the others. For example, intraperitoneal administration achieved higher systemic concentrations in comparison to other routes (Malik et al., 2017). This takes us to a momentous issue; phage stability. It means that a phage should maintain its infectivity without any loss in titer from the time of manufacture and storage passing by administration to the moment of reaching the infection site. A score of variables determine this stability (Slopek et al., 1983). For the most part, phages are protein in nature. So each of temperature (Briers et al., 2008), pH (Briers et al., 2008; Knezevic et al., 2011), ionic strength (Briers et al., 2008) and shear stress (Leung et al., 2016) are inclined to influence them in a way that may even cause denaturation. Exposure to these factors during isolation, storage and even after administration should be diligently considered (Malik et al., 2017). For storage, different phages were found to be stable in media like Trypticase Soy Agar and Brain Heart Infusion at 4^°^C, other temperatures have been tested e.g.: -20^°^C and -80^°^C in the presence of a cryoprotectant like glycerol (Clark, 1962). Stabilizers like trehalose and sorbitol can also be considered for longer storage periods (Hubálek, 2003). In every way, a little reduction in titer should be expected (Dedrick et al., 2019a). A neutral pH is required even after administration (Gordillo Altamirano and Barr, 2019). For this reason, we should seek innovatively protective delivery systems for phages especially those considered for an oral route. Novel techniques like encapsulation of phage particles in nanocapsules (Malik et al., 2017) proved its worth in maintaining its efficacy during storage, and after administration (González-Menéndez et al., 2018). In a fascinating study, encapsulation even contributed to prolonging circulating phage time eight-folds by eluding antibodies (Singla et al., 2015; Chadha et al., 2017). The rational design of stimuli-responsive dosage forms is going to be the focus of attention in near future. It may allow for targeting phages to the infection site making use of the physicochemical properties in that place (Malik et al., 2017).

The Second arm to phage kinetics is actually disregarded most of the time. It is simply interested in the key parameters governing the virion-bacterium interaction and so, the phage titer throughout the infection time  (Leung and Weitz, 2017). They are specific for each duplet and might show difference according to circumstances; whether in-vivo or in-vitro. Due to their influence, they deserve to be the quest of more studies. The most important among these parameters are:

a. Adsorption rate of phage particles to the bacterial cell. Different reaction kinetics equations were devised to describe the binding of phages to their host cells. However, multiple factors influence the process, like: agitation, temperature, bacterial cell size and its growth phase at the time of attachment, the density of binding receptors on its surface, the presence of ions, and organic compounds; for example tryptophan is essential for T4 phage attachment. Considering all this, it is highly unlikely for a single equation to be able to accurately predict bacteriophage adsorption rate in all phage-host duplets. That being said, Krueger’s postulation long ago that phage adsorption follows first-order reaction kinetics remains the one applied the most today (Kropinski, 2009; Storms and Sauvageau, 2015).
b. Multiplicity of infection (MOI): defined as number of infective virions per target bacterium during infection. It varies according to the available densities of both: host cells and phages, and is also related to the time taken for adsorption to occur and the total time allowed for contact between the target host and its phage (Abedon, 2016).
c. Latent period: the time taken to complete one lytic cycle inside a bacterial cell. This period is determined primarily by phage holins, the proteins that regulate the digestive activity of the endolysins. It is also affected by the host cell physiology as it gets faster whenever a high concentration of healthy host cells is available (Abedon, 1989; Wang et al., 1996; Abedon et al., 2001).
d. Burst size: number of newly formed phages liberated per bacterium after cell lysis.
Both terms latent period and burst size are determined by the physiology of the infected bacteria, the phage holins as they control the timing of the lysis, and the infection conditions (Abedon et al., 2001).
e. The initial count of both bacteria and phage at the infection site. This determinant might be a bit controversial because high yet optimal initial concentration of the phage is required. A phage count that is too high in terms of MOI may lead to bacterial lysis only *via* the enzymatic action of the phage lysins once the phages are attached to their receptors, without virions even accessing the cell and so the productive infection of bacteriophages comes to a halt; generating no more viruses to invade the rest of the pathogenic population (Delbrück, 1940). This was described by Delbruck as “lysis from without”; a phenomenon that is defined as: “phage-induced bacterial lysis that is directly associated with phage adsorption rather than with factors that are synthesized within the subsequently lysing bacterium” (Abedon, 2011b).
f. Finally, the bacterial host growth rate. According to the recent work by Nabergoj et. al., bacterial growth rate significantly affects the previous parameters, even though it depends on the host-phage system in question (Nabergoj et al., 2017).

Obviously, these parameters are not easily defined, and are usually screened by the old established factors. So, resolving the ambiguity surrounding them is required if phages are to settle as antibacterial drugs (Malik et al., 2017).

## Marked Real Life Experiences Involving Bacteriophages

Although antibacterial resistance is a powerful incentive to hit on phage therapy as a potential solution, there is a deficit in human trials of therapeutic phage application. The reasons are manifold: safety concerns, phage peculiarities, absence of standard application protocol and the necessity of phage thorough characterization. All these are hurdling the progress of clinical phage trials. So, there is a need for better organization of human trials on phages and improving the reporting of their data and results (Vandenheuvel et al., 2015; Abdelkader et al., 2019). However, some cases testing for phage safety and efficacy have been reported.

### PhagoBurn-Cornerstone in Phage Therapy

Although the results were frustratingly less than expected, this famous “PhagoBurn” clinical trial plausibly deserves to be described as a climacteric landmark in the history of phage therapy. PhagoBurn was initiated in 2013, Europe, under the title of “Evaluation of phage therapy for the treatment of *Escherichia coli* and *Pseudomonas aeruginosa* burn wound infections (Phase I-II clinical trial)” (Lin et al., 2019). It was the first time ever for three different national regulatory agencies to reach a consensus on trying a phage cocktail for human therapeutic purpose. The three agencies were ASNM-France, AFMPS-Belgium and Swissmedic-Switzerland. Adding to its specialness, this multi-centered prospective trial used a phage cocktail typically manufactured according to the requirements specified by supervisory bodies: Good Manufacturing Practice (GMP) and Good Clinical Practices (GCP) (Sarker et al., 2016). Unfortunately, this pioneering project came to a close in 2017 because of slow progress in the therapeutic objectives (Jault et al., 2019). Compared to the standard treatment, the applied phages took more time to reduce the infectious burden, so, the use of higher titers of phages and in-vitro testing of bacterial isolates susceptibility to the phages prior to resuming therapy are strongly recommended for future trials. All these trials perhaps are paving the way for us to re-implement bacteriophages as a Trojan horse in front of the constantly arising superbugs.

### Cystic Fibrosis (CF)

CF is a distinctive disease among the genetic disorders and is becoming increasingly problematic. It is caused by a mutation in CFTR (cystic fibrosis transmembrane conductance regulator) gene (Davies et al., 2007). It might affect several organs such as liver, intestine and pancreas. Nevertheless, it is most troublesome in the lung (Donlan and Costerton, 2002). It is characterized by massive impairment in the mucociliary function (Davies et al., 2007) such that; excessively viscous mucous (Donlan and Costerton, 2002) hinders the efficient clearance of invading organisms and induces a superfluous inflammatory reaction (Davies et al., 2007).This confers liability to difficult-to-cure chronic infections (Donlan and Costerton, 2002). A few particular organisms are involved including *Haemophilus influenzae*, *Staphylococcus aureus* and *Pseudomonas aeruginosa* (Donlan and Costerton, 2002). However, the most critical organism is the so disreputable *Burkholderia cenocepacia*. It is an opportunistic organism yet armored with so many virulence factors. In CF patients, it causes cepacia syndrome, a kind of infection accompanied by a huge drop in lung function and usually complicated with fatal sepsis. In our context, it is becoming stubbornly resistant to antibiotics and usually costs the patient his life (Scoffone et al., 2017). Based on the foregoing, it is a dire need to appraise bacteriophages effective against *Burkholderia cenocepacia* (Carmody et al., 2010). In a study testing the effectiveness of phage BcepIL02 against *Burkholderia cenocepacia*-induced lung infection in an animal model, their results demonstrated that bacteriophages could be a sensible solution in the future. Another research article reported that a combination of phage and certain antibiotics can be a viable option while confronting this microorganism (Kamal and Dennis, 2015). Uniquely, in the period between 2007 and 2010, eight CF patients in Tbilisi received aerosolized bacteriophages along with other medicines. A reduction in the count of *Pseudomonas aeruginosa* residing in patients’ sputum was significantly noticed (Hraiech et al., 2015).This study may be a flicker to pluck up courage and try out the same with *Burkholderia cenocepacia.*

### MRSA (Methicillin-Resistant *Staphylococcus aureus*)

*Staphylococcus aureus* had the distinction to be the first documented experience of phage therapy. It was applied for treating skin infections caused by this organism (Sulakvelidze et al., 2001; Pizzorno and Murray, 2012). MRSA is characterized by low susceptibility to oxacillin and many other antibiotics (Siddiqui and Koirala, 2020), making it so hard to cure. Nowadays, it is becoming a special concern because of its pervasiveness in daily life communities like schools and nurseries (MRSA | CDC, 2019). In addition, it is one of the foremost causes of nosocomial infections usually associated with serious and sometimes lethal complications (Siddiqui and Koirala, 2020). A phage product of reasonable spectrum against multiple staphylococcal strains was previously used in the United States under the name of *Staphylococcus* Phage Lysate-(SPL), however its human use was abruptly proscribed by FDA in the 1990s owing to regulatory issues (Sulakvelidze et al., 2001; Pizzorno and Murray, 2012; Besser et al., 2018; Mitchell and Simner, 2019). Just a decade earlier in the Eliava Institute, phage was systemically used against MRSA and proved efficacy. In Georgia, a complementary approach is the use of anti-MRSA phages as a disinfectant for infection control in operation rooms (Abedon et al., 2011a). Moreover, in one recent study, safety of phage therapy was tested in some serious *S. aureus* infections in humans. The researchers concluded that their phage was completely safe and resulted in no adverse effects (Petrovic Fabijan et al., 2020). In the same context, recently an FDA expanded the access to an IND preparation of three phages (AB-SA01) fabricated by the phage manufacturing company: AmpliPhi (Jacobs et al., 2019); together with a specific antibiotic regimen were successfully used for the management of an elderly man suffering from staphylococcal sepsis along with prosthetic valve endocarditis. Worthy of mention; this has been the first staphylococcal prosthetic valve endocarditis case cured with bacteriophage applied intravenously (Gilbey et al., 2019).

### Mycobacterial Infections

Infections caused by mycobacterial species have always been a perturbing medical issue. Besides tuberculosis (TB), they can cause a lot of invincible diseases like leprosy and Buruli ulcer (Azimi et al., 2019). Being intracellular organisms, they are difficultly accessible when inside the human body. Also, they are characterized by a sturdy, hard-to-penetrate cell wall structure; with mycolic acid linked to the peptidoglycan *via* an arabinogalactan (AG) junction. A few antibiotics are regularly applied for treatment of mycobacterial infections like: isoniazid, rifampin, pyrazinamide, ethambutol and streptomycin (Nienhuis et al., 2010). Despite of their efficacy, they are usually administered for lengthy regimens, rendering the patient susceptible to several toxicities; ototoxic, nephrotoxic and hepatotoxic effects might ensue (Dedrick et al., 2019a). In 2017, WHO alarmingly announced that resistant strains of *Mycobacterium tuberculosis* have emerged (Sinha et al., 2020). Altogether, obtaining bacteriophages active against these organisms and capable of working complementarily to antibiotics is appealing. Indeed, around 10,000 mycobacteriophages have been isolated using *Mycyobacterium smegmatis*-a fast-growing, non-virulent species-as a host (Hatfull, 2014). Unfortunately, little is known about the nature of these phages and their host range; whether they can be effective against other mycobacterial species or not (Dedrick et al., 2019b). However, some of them have been tested against the virulent species and proved preliminary efficacy. For instance, a young patient suffering from cystic fibrosis complicated by dissemination of *Mycobacterium abscessus* infection after a lung-transplantation was then subjected to the intravenous administration of a cocktail formed of three phages namely: Muddy, BPs33ΔHTH-HRM10, and ZoeJΔ45; each of them was originally isolated against *M. smegmatis*. After six weeks of treatment, this resulted in substantial clinical improvement presented as closure of the sternum wounds and resolution of the skin nodules with no side effects observed. Uniquely, this was the first study applying phage therapy for mycobacterial infections in human (Tseng et al., 2019). Another example is related to *Mycobacterium tuberculosis*. Many techniques have been strenuously studied in order to control tuberculous mycobacterium both in the air and on surfaces and so, reduce the infection risk. In the recent work performed by Tseng et al., a bacteriophage was isolated from soil using *M.smegamatis* that is analogous to *M.tuberculosis* for safety considerations. A calculated concentration of *M.smegmatis* was released in the study chamber followed by the spraying of phage aerosol preparation. The results were reduction in the number of air-borne *M.smegmatis* and bactericidal effect onto the surface of agar plates contaminated with the same organism. These observations suggest that phage aerosol could be an auspicious tool for controlling tuberculosis transmission and also hold a promise for phage efficacy if applied therapeutically in this regard (Payne et al., 2009). Along the same line, a mycobacteriohage-D29, proved to encode for an additional lysin: lysin B; an esterase that breaks the bond joining mycolic acid to the arabinogalactan (Fraga et al., 2019; Puiu and Julius, 2019). A mouse footpad model was designed to examine the effect of a recombinant version of this special enzyme onto a certain non-tuberculous mycobacterial infection; Buruli ulcer. It is a serious disease of the skin, caused by *Mycobacterium ulcerans* and leads to several necrotic ulcers. It also represents the third most frequent mycobacterial infection. The result suggested that Lysin B has a substantial protective effect and hinders further bacterial multiplication (Górski et al., 2009).Two problems are facing the idea of treating mycobacterial infections using bacteriophage. First: bacteriophages lack the ability to make their way through the human cell membrane and hence, unable to reach these intracellular pathogens. This was innovatively tackled by using *M.smegmatis* as a delivery vehicle of bacteriophage TM4 that managed to transfer it into the macrophages infected with TB and eventually led to a considerable drop in TB titre (Markoishvili et al., 2002). The second issue, that is yet to be tackled, is that the layered formation of granuloma -a hallmark of tuberculous infections- may further hinder the ingress of phages to the target cells. Downright, in spite of the movement in favor of phage therapy for mycobacterium species, thorough experimentation is still required in order to apply it successfully and safely (Weber-Dabrowska et al., 2003).

### Emergency Investigational New Drug (eIND)

Up till now, there is no phage preparation approved by FDA for human therapeutic use (Principi et al., 2019). And for this to happen, both safety and efficacy of bacteriophage therapeutics must prove abidance by the rules currently governing the licensure of a new drug. Consequently, their current use is restricted to the so-called “compassionate use” (the use of a new, unapproved drug to treat a seriously ill patient when no other treatments are available). Sometimes, this works as a rescue therapy for difficult-to-cure infections. Not so long ago, particularly in 2006, a glimmer of hope had arisen when a phage cocktail effective against Listeria was granted the FDA approval as the first antimicrobial phage preparation for controlling the food-borne pathogens. This was a breakthrough in food microbiology. Under the commercial name of “ListShield™”, it was the first phage preparation to be classified as GRAS.(Generally Regarded As Safe) (Bren, 2007; Perera et al., 2015; Gutiérrez et al., 2017; Yang et al., 2017). Again, only two years ago, the same company “Intralytix” received FDA clearance to launch phases I and IIa clinical trials to test a proprietary set of bacteriophages for treatment of human inflammatory bowel diseases (IBD). According to the company’s official website: The project aimed to test the feasibility and efficacy of the bacteriophages in controlled human clinical trials. This is one of the first full-blown Investigational New Drug (INDs) approved for phages by the FDA, and the first ever for targeting adhesive invasive E. coli (AIEC). This paves the way for more-to-come clinical trials (Intralytix, Inc), therefore, a scrupulous compilation of regulatory rules has to be firmly established to allow the introduction of phages into clinical practice (Plaut and Stibitz, 2019; Bretaudeau et al., 2020).

In 2017, Schooley et al. reported on a deteriorated case of an elderly male diabetic patient. He had been suffering from necrotizing pancreatitis that further complicated by a refractory intra-abdominal Acinetobacter baumanii infection. An eIND phage cocktail of nine different bacteriophages active against the patient’s specific isolate was eventually commenced as a rescue therapy. Fortunately, it resulted in microbial clearance of the infection accompanied by clinical resolution (Schooley et al., 2017).

On a regrettable incident, the death of a 2-year-old infant with a complex of DiGeorge syndrome and a relentless Pseudomonas aeruginosa bacteremia has occurred lately, mostly because of cardiac failure. However, prior to this, an eIND phage cocktail had been secured, and proved to cause microbial eradication from the child’s blood (Duplessis et al., 2018). These trials should evoke more endeavors towards a broader application of phage INDs.

### What Is Next?

Some of the reservations concerning the application of phage therapy in a traditional way stem from its very nature as an evolvable entity whose biology is yet to be completely identified. As a result, there is now a growing inclination towards the tactics that can convert phages from purely biological organisms into drug-like agents. An outstanding study has addressed this problem lately utilizing the method of photothermal ablation. “Phanorods” are units of phages attached to nanorods formed of gold. These were designed to specifically target the problematic bacterial cells. Using a method of infrared irradiation, the gold nanorods will release energy enough to destroy both the bacterial cells and the phages themselves in location, decreasing the gene transduction risk that might accompany therapeutic phages if remaining alive. This paves the way for a platform of controlled phage therapy.

### Phage and COVID-19

As of October 2020, Górski et al. pointed out a timely hypothesis. They postulated that phage therapy could serve as an adjunct in the management of the viral sepsis caused by the latest pandemic; COVID-19 (Górski et al., 2020). They founded their assumption on multiple analogies. Among them are the following:

-T4 phage proved to have a preventive action against the infection with human adenovirus (Adv) (Miedzybrodzki et al., 2005). It led to inhibition of the viral adsorption to human pulmonary epithelial cells and hence, viral replication in the lung as well (Przybylski et al., 2015; Przybylski et al., 2019; Bichet et al., 2020).
-Phages usually downregulate the production of reactive oxygen species (ROS) induced by bacteria (Międzybrodzki et al., 2012). Since lung cells actively infected with SARS-CoV-2 show high levels of ROS, phages might help alleviate this inflammatory concomitant (Lin et al., 2006; Kim et al., 2020).
-*In-vitro* results have shown anti-apoptotic effect of certain phages on the epithelial cells of the human airway (Trend et al., 2018). This could be exploited to mitigate the viral-induced pulmonary apoptosis (Lin et al., 2006; Wang et al., 2020).
-Also, T4 phage is known to enhance the expression of a peptide encoded by the human beta defensin 2 gene (hBD2) in the epithelial cells (Borysowski et al., 2020). This is responsible for decreasing viral replication and prompting the anti-viral innate immune response. It dramatically reduced the invasion of human respiratory syncytial virus (HRSV) into human lung epithelial cells (Kim et al., 2018). Likewise, this property could be harnessed to ultimately result in the containment of COVID-19 infection.
-Studies propose that platelets contribute to the dissemination of COVID-19 inside the human body *via* its binding to alphaIIb/beta3 integrin on the platelet membrane. Surprisingly, this integrin can bind to a KGD (Lys-Gly-Asp) sequence motif displayed by T4 phage as a capsid protein. This could be utilized so as to enable this phage to interfere with COVID-19 binding to platelets.

These support the hypothesis that phage therapy could be applied not only as antibacterial but also for anti-viral purposes (Manne et al., 2020). For this assumption to prove true, experimental trials are promptly needed.

## Bacteriophage: A Jack of All Trades?

Apart from using phage as an antibacterial, it may provide a solution to other worrying medical conditions too. Thanks to their exceptional ability to cross the adamant shield of blood-brain-barrier (BBB), a great hope has arisen for those suffering from nervous system disorders and degenerative diseases like Parkinsonism and Alzheimer (Lehti et al., 2017). In the commendable work of a previous study (Messing, 2016), bacteriophage M13 was genetically designed to display antibodies active against misfolded proteins like β-amyloid and α-synuclein; the hallmark of such diseases. In addition, phage could be exploited into the domain of cancer therapy *via* one of two approaches. The first: up-regulation of the patient immunity, enabling it to fight hard against this enduring disease (Weber-Dabrowska et al., 2001). In their work, Górski et al. noticed an increase in the level of certain immune substances like anti-tumor cytokines as a result of the exposure of immune cells to some unusually attractive phage capsid proteins (Weber-Dabrowska et al., 2001; Budynek et al., 2010). Second, using the phage display technique, modified phage M13 active against *Fusobacterium nucleatum* was tested in an attempt to treat colorectal cancer (CRC) and it played a tripartite role. First, it helped in clearance of *Fusobacterium nucleatum*, the first suspect in suppressing the host’s antitumor immune response in front of CRC. Also, it acted as an apt targeted delivery system since it was decorated with silver nanoparticles entrapping certain chemotherapeutic drugs. Third, being so embellished with these nanoparticles, it succeeded to further stimulate the immune system (Dong et al., 2020). It is reasonable here to stress on the point that being inherently specific, phages might be so beneficial in targeting the bacterial infections associated with certain types of cancer like *Helicobacter pylori* in gastric cancer and *Salmonella enterica* in biliary cancer (Chang and Parsonnet, 2010). And as a side asset, they could be loaded with anticancer drugs as well. This would lead to targeted delivery of chemotherapeutic anticancer drugs, helping in limiting their deterrent adverse effects. (Bar et al., 2008).

Phages can also be used as delivery vehicles for mitigating antibiotic side-effects. “Antibacterial nanomedicines”, this is how bacteriophages were described by Yacoby et al. in 2007. Back then, they managed to complex chloramphenicol antibiotic molecules to bacteriophages. This provided two great advantages. First, it conferred specificity to the antibiotic molecules, only interacting with their target pathogens and hence radically reduced its debilitating side effects by avoiding contact with sound human cells. Second, concentrating the antibiotic at a certain location enhanced its potency against bacterial cells (Yacoby et al., 2007).

**Figure 3:**
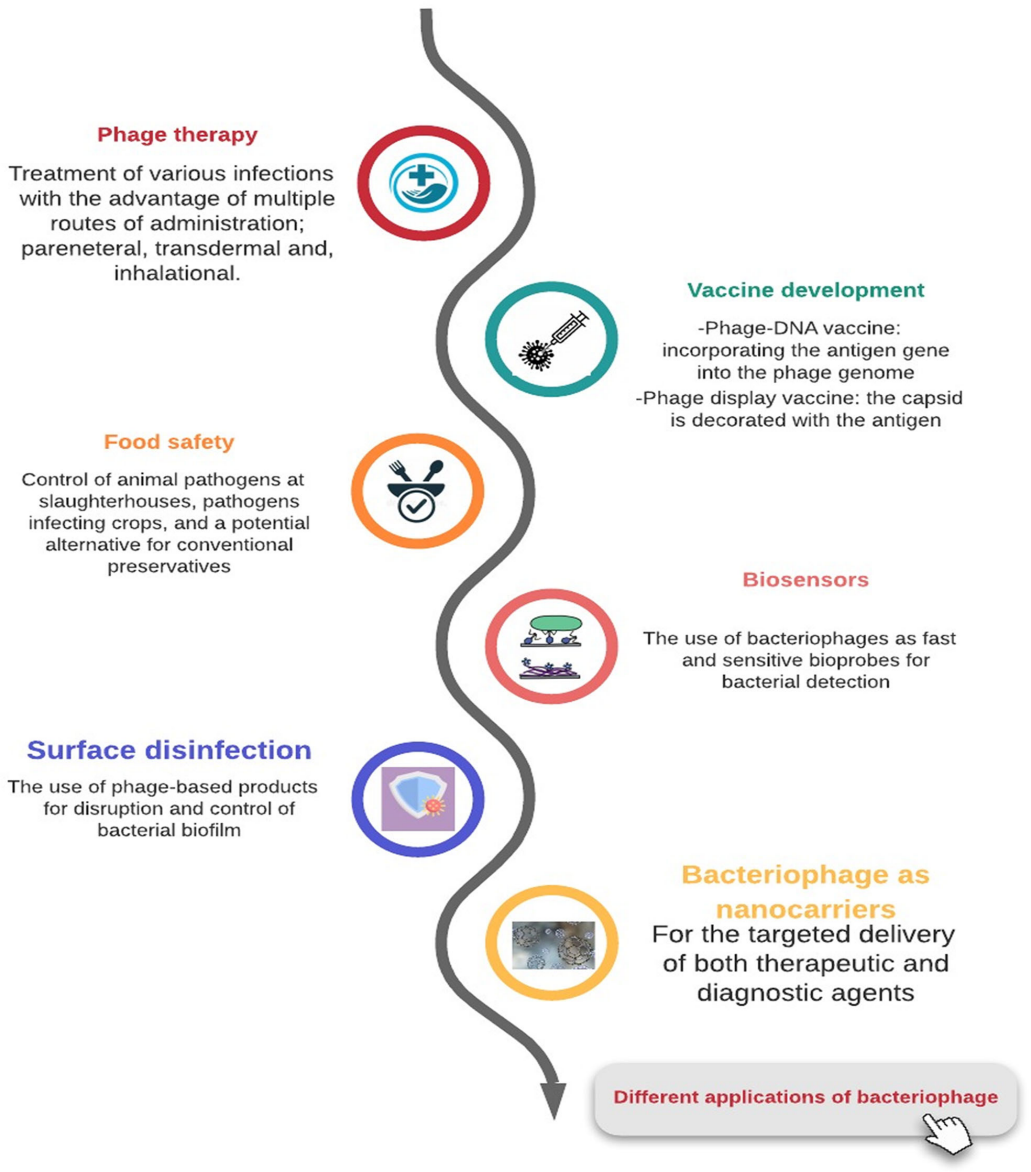
Various applications of bacteriophages in different fields.

One more interesting application of bacteriophage is related to the sector of food safety. The undesirable effects of the chemical food preservatives are already well-known (Sharma, 2015). This spurred the searching for a replacement, like biopreservation which is currently being considered (Moye et al., 2018). Bacteriophage possesses much of potentiality to healthfully prevent bacterial contamination of food (Dewey-MattiaETAL; Oluk and Karaca, 2018). Relevantly, a recent study showed optimistic results for the prophylactic use of bacteriophages as probiotics against some pathogenic strains (Mai et al., 2010). **Figure 3** is presenting some of the various phage applications in different fields (Harada et al., 2018).

Furthermore, and without a limiting clause, bacteriophages were put to test as an alternative to antibiotics for controlling pathogens in the fields of both agriculture and animal farming. Phages proved to be of benefit in the fields of poultry farming (Smith and Huggins, 1983; Atterbury et al., 2007), pastoral farming, and even in pisciculture. They may be able to rid us from the economic burden posed by the not-so-efficient methods used for fighting animal diseases (Nakai et al., 1999; Johnson et al., 2008).

Now, after digging deep in the life-world of these little creatures; it was a chance to highlight their multifaceted potential, discover how bacteriophages were long-forgotten, cover some of their various applications and most importantly shed the light on the role they can play in calling the danger of bacterial resistance to a halt. It is time to embrace a redeeming stance and go after the untapped potential of phages in different arenas. However, for phages to become a reliable mainstream drug, it is crucial to create an educative climate where public acceptance of using “viruses” in many life aspects, is possible. This could happen only if all the parties concerned: scientific communities, mass media workers and legislators; show true interest and cooperate to turn this idea into reality. A great challenge while promoting for such idea is being truthful to the public audiences. Scientific integrity should always come in the first place, transferring the full picture with neither underestimation nor exaggeration (Gordillo Altamirano and Barr, 2019).

## Conclusion

Life is better understood backwards yet must be lived forward. From this standpoint, our article is calling out to hold in high regard all the expanding literature and valuable knowledge that have been piling-up for almost a century regarding bacteriophage, its multipurpose potentialities and most importantly, its therapeutic forte. Being on the cusp of post-antibiotic era; phages could be our allies, and enactment of phage therapy might be a fortunate trailblazer in the global fight against bacterial resistance. Certainly, several question marks still hover around bacteriophages, like their ability for horizontal gene transfer, the interaction dynamics with the human microbiome, and the different findings on their immunomodulatory effects; all these need to be tackled prior their application for therapy. Also, this corroborates the fact that no single strategy can overcome the danger posed by antibiotic resistance. Hence, the integration of phage therapy, their lytic proteins, and engineered phages, while keeping the efforts addressing the antibiotics’ problems; is of utmost importance facing such a huge issue.

## Author Contributions

IMA, GSH and KMA wrote the paper in its final format. All authors contributed to the article and approved the submitted version.

## Funding

This research was funded by Research Center for the Advanced Materials Science (RCAMS) at King Khalid University under grant number RCAMS/KKU/016-20.

## Conflict of Interest

The authors declare that the research was conducted in the absence of any commercial or financial relationships that could be construed as a potential conflict of interest.
